# Introduction to the Quantitative Analysis of Two-Dimensional Fluorescence Microscopy Images for Cell-Based Screening

**DOI:** 10.1371/journal.pcbi.1000603

**Published:** 2009-12-24

**Authors:** Vebjorn Ljosa, Anne E. Carpenter

**Affiliations:** Imaging Platform, Broad Institute of MIT and Harvard, Cambridge, Massachusetts, United States of America; Whitehead Institute, United States of America

## Introduction

Modern automated microscopes collect digital images at an astonishing pace. Automated image analysis can measure biological phenotypes quantitatively and reliably, and has therefore become a powerful tool for probing a wide variety of biological questions using microscopy. In this tutorial, we acquaint biologists with this important computational field and introduce some basic principles of image analysis, using typical strategies for two-dimensional images of cultured cells in high-throughput screens as the primary example.

## Why Use Automated Image Analysis?

Microscopy is one of the foundational tools of biology, and researchers have for centuries relied on their own visual systems to interpret what they see. Although examining tens of thousands of samples by eye is tedious, biologists are often highly motivated to invest the effort in order to discover samples of interest or annotate large sets of chemically or genetically perturbed samples. One impressive example is a genome-wide RNA-interference screen for dozens of phenotypes, where extensive manual annotation of more than 40,000 movies of early embryogenesis in *Caenorhabditis elegans* uncovered the detailed involvement of hundreds of genes in development [1]. Annotation of such complex and varied phenotypes is beyond the capabilities of current computer software.

Yet there are many cases where scoring visual phenotypes with a computer is highly attractive. The most obvious advantage of automated image analysis is speed, especially now that automated microscopes can capture images faster than a human can examine them. This enables experiments on an entirely different scale than before; for example, an automatically analyzed microscopy screen of the human genome by RNA interference (more than 300,000 images) recently revealed many classes of mitosis-essential genes in multiple phenotypic categories [2]. As a second example, counting dozens of DNA-damage-induced foci in each of hundreds of cells in each of tens of thousands of images would simply be impossible by eye; yet automated image analysis enabled such a screen to identify regulators of DNA-damage responses (Scott Floyd, Michael Pacold, Thouis R. Jones, Anne E. Carpenter, and Michael Yaffe, unpublished data).

Often the goal of automated image analysis is simply to replicate a human's observations with less labor. There are other substantial scientific benefits, however: automated image analysis can yield objective and quantitative measurements, thereby enabling the capture of subtle differences among samples as well as statistical analysis and systems-biology research on the data. In the case of hundreds of phenotype-relevant genes or chemicals discovered in a single screen, the quantitative measurement of multiple cellular phenotypes enables those samples to be sorted into distinct subtypes for further analysis and characterization, as has been done recently for mitotic-spindle defects [2] and defects in cytokinesis [3]. Researchers have also identified situations where automated image analysis can “see” phenotypes invisible to humans. For example, researchers typically cannot distinguish cells in the G1 phase of the cell cycle from those in G2 by looking at images of DNA-stained cells, but automated algorithms can do so by quantifying the fluorescence intensity of the DNA in each nucleus [4]. Computers have also been able to distinguish the subtle differences between localization patterns that seem identical to a human investigator [5].

## Educational Article Overview

Although learning about image analysis can be daunting, an understanding of the basics is critical for successful analysis. The effort will pay off whether planning a high-throughput screen, a time-lapse experiment, a systems-biology project, or just analyzing a small-scale experiment quantitatively.

**Figure 1:**
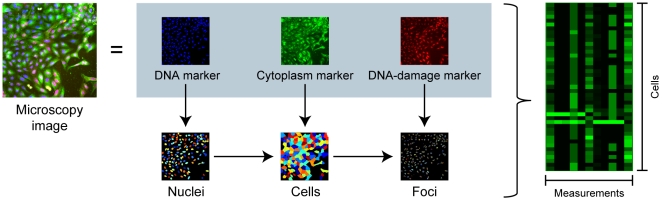
Overall image analysis workflow for a typical experiment. First, variations in illumination and staining are corrected. Nuclei are identified by thresholding, then used as seeds to identify cell edges. Finally, DNA-damage foci are identified. Schematic data shown, based on image courtesy of Scott Floyd, Michael Pacold, and Michael Yaffe. Colors of nuclei, cells, and foci are arbitrary.

In this article, we give an overview of the basic concepts of automated image analysis, using simple techniques that are useful for two-dimensional fluorescence images of cultured cells as an example. We walk through a typical image-analysis workflow (Figure 1), explaining the basic concepts, methods, and software for determining which pixels in an image belong to each cell or cellular compartment and measuring interesting properties of these objects, as well as alternative approaches for images in which identifying each object is infeasible.

Throughout this tutorial, we will use the example of a cell-based fluorescence microscopy assay for DNA-damage regulators (Figure 1). The goal in this assay is to identify samples where cells show an unusually strong or unusually weak response to DNA damage by counting the number of DNA-damage-induced foci per cell. The foci are labeled by an antibody that recognizes the phosphorylated form of a protein that responds to DNA damage. We and our collaborators have used this assay to screen chemical compounds and genes (using RNA interference) in human cells and *Drosophila melanogaster* cells to identify regulators of DNA-damage-response pathways (Scott Floyd, Michael Pacold, Thouis R. Jones, Anne E. Carpenter, and Michael Yaffe, unpublished data).

This is only an introductory taste of how image analysis works, exemplified by one particular application area. We do not attempt a comprehensive review of biological image analysis but instead point the reader to excellent resources in the field (see Box 1). These resources are more comprehensive review articles that cover the latest developments in the broader world of biological image analysis, including analysis for three-dimensional image stacks, time-lapse images, analysis of whole organisms, and imaging modalities like brightfield microscopy, differential-interference-contrast imaging, electron microscopy, and biomedical imagery (MRI and PET scans of humans or model organisms, for example).

**Box 1. Resources for further exploration**

The following suggestions do not represent a comprehensive listing. Rather, the sampling of resources listed here should guide the interested reader to begin exploring the field of image analysis for microscopy.

**Review articles:**

- Biological image analysis in general, especially geared towards biologists [40]
- Biological image analysis in general, especially geared towards computer scientists [41]
- Large-scale or high-throughput microscopy screening of chemical or genetic perturbants [42]–[50]
- Image-acquisition pitfalls [51]
- Infrastructure and informatics for high-throughput image acquisition and analysis [52]–[55]
- Imaging modalities [56]
- Thresholding methods [57]
- High-throughput microscopes [58],[59]
- Time-lapse and three-dimensional biological image analysis [25],[60]
- Electron microscopy image analysis [61],[62]
- Confocal image acquisition [63]
- Image acquisition and analysis for colocalization studies [64],[65]
- Image analysis for characterizing fluorescence localization [26]
- Image acquisition and analysis for nuclear substructures [66]
- Online resources for biological image analysis, including software [67]

**Societies/conferences:** Major conferences and societies covering biological image analysis are the BioImage Informatics conference (http://www.bioimageinformatics.org), the International Society for Advancement of Cytometry (ISAC, http://www.isac-net.org/), and the Society for Biomolecular Sciences and its Data and Image Analysis Special Interest Group (http://www.sbsonline.org). Also useful is the Microscopy Society of America (http://www.msa.microscopy.org) and the Optical Society of America (http://www.osa.org). With a stronger computer science perspective are the workshops on Microscopic Image Analysis with Applications in Biology (http://miaab.org), IEEE International Symposium on Biomedical Imaging (ISBI) conferences (http://biomedicalimaging.org), and SPIE (http://spie.org).

**Training/workshops:** Opportunities for learning about microscopy and image analysis include workshops and tutorials affiliated with the conferences and societies listed above, as well as companies that offer training for their microscopes and software. Other courses available include those organized by the Marine Biological Laboratory at Woods Hole (http://www.mbl.edu/education/courses/special_topics), Cold Spring Harbor Laboratory (http://meetings.cshl.org), John Russ (http://www.drjohnruss.com/courses.html), and EMBL/EMBO courses.

**Websites/discussion groups:** The High Content Imaging Google group (http://groups.google.com/group/highcontent) provides listings of software, hardware, conferences, and resources for the field. Helpful tutorials about image analysis and image acquisition have been collected at the “Molecular Expressions” Optical Microscopy Primer (http://micro.magnet.fsu.edu/primer/index.html).

**Journals publishing image-analysis techniques:** *Cytometry*, *Journal of Microscopy*, *Microscopy Research and Technique*, *Microscopy and Microanalysis*, *Microscopy Today*, *Nature Photonics*, *Journal of Biomolecular Screening*, *Bioinformatics*, *BMC Bioinformatics*, *Neuroinformatics*, *Journal of Biomedical Informatics*, *IEEE Transactions on Pattern Analysis and Machine Intelligence*, *International Journal of Computer Vision*, *Proceedings of SPIE*, *IEEE Transactions on Medical Imaging*, and *IEEE Transactions on Image Processing*. Primary research is also published in the proceedings of the following conferences: IEEE International Symposium on Biomedical Imaging (ISBI), International Conference on Medical Image Computing and Computer Assisted Intervention (MICCAI), International Conference on Image Processing (ICIP), IEEE Computer Society Conference on Computer Vision and Pattern Recognition (CVPR), IEEE International Conference on Acoustics, Speech, and Signal Processing (ICASSP), and International Conference on Computer Vision (ICCV).

**Books:** A helpful overview of many issues in biological image analysis is the book *Microscopic Image Analysis for Life Science Applications*, by Rittscher, Wong, and Raghu [68], which covers types of microscopy, probe selection, and image-analysis techniques relevant for biological images. *The Image Processing Handbook*, by John Russ [69], and *Digital Image Processing*, by Gonzales and Woods [70], are helpful overviews for image analysis and image processing in general.

**Image analysis software:** There is no one-size-fits-all software package for all goals in biological image analysis. Different software is geared for different applications (e.g., time-lapse, three-dimensional, and particular cell types like neurons). The Internet Analysis Tools Registry (http://www.cma.mgh.harvard.edu/iatr/display.php?spec=all) and The American Society for Cell Biology (http://cellbase.ascb.org/research.html#Vendors) provide guides to software. For the workflow used as an example in this article (two-dimensional, high-throughput images), the following software are some examples (note that this list is not comprehensive):

*Commercial vendors*: MetaMorph, ImagePro Plus, ThermoFisher Cellomics, GE InCell, PerkinElmer Evotec Opera, Molecular Devices MetaXpress, BD Pathway (Atto), CompuCyte, TTP Labtech Acumen Explorer, and Definiens Cellenger. Most of these are high-throughput microscope vendors and license fees range from thousands to tens of thousands of dollars per year in addition to the cost of the microscope. These software packages generally have polished user interfaces and are well integrated with the microscope hardware and image-acquisition process. Software like MATLAB is available for programmers.

*Open-source software projects*: ImageJ [71] has a large user community that has produced hundreds of plugins for different applications. It is also possible to write or record macros to automate tasks, or to use ImageJ as part of custom-written analysis programs. Our own group has created CellProfiler [72],, an open-source software package that is tailored for automated high-throughput image analysis (http://www.cellprofiler.org). The FARSIGHT project (http://www.rpi.edu/ roysab) is developing multi-dimensional image-analysis tools for microscopy data. For developers, ITK (http://www.itk.org) is an open-source library of algorithms and software tools for image analysis and VTK (http://www.vtk.org) is an open-source system for 3D computer graphics, image processing, and visualization.

## Image Analysis Basics

A digital camera attached to a microscope divides the field of view into a grid of *pixels*. The intensity of the light absorbed by a pixel is recorded as that pixel's numerical value. The digital image that the computer has to work with for image analysis, then, is a grid of numbers, each of which indicates the intensity of light in a small part of the field of view. If different channels are imaged (e.g., for different fluorescent wavelengths), there will be one such grid for each channel. The role of image analysis is to transform these grids of numbers into measurements of biological relevance, such as the number of cells and the number of DNA-damage-induced foci. As we will later see, a wide diversity of phenotypes of biological interest can be measured from images, including the amount of DNA in each nucleus, the degree of cytoplasm-nucleus translocation, and the presence of biologically relevant morphologies.

## Identifying Image Foreground

**Figure 2:**
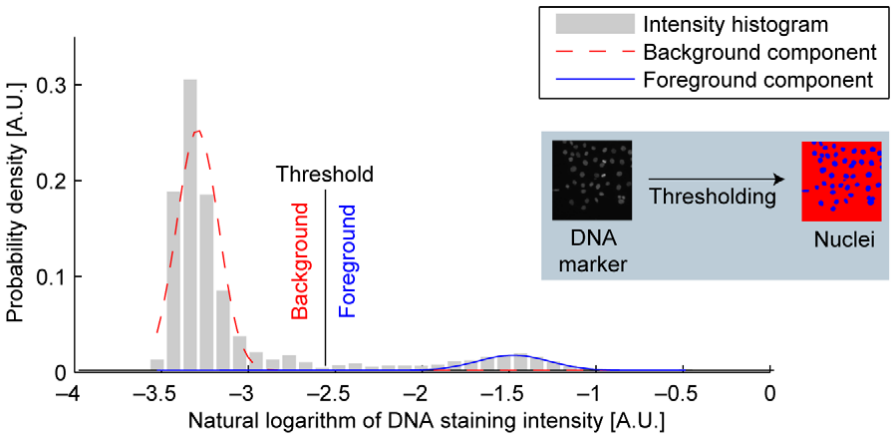
Thresholding by mixture models. Mixture models derive a threshold from two density functions (one for the background, one for the foreground) fitted to the distribution of intensities in the image. Units are arbitrary. Original image from project described in Moffat et al. [80].

The most challenging part of image analysis is usually determining which pixels in the image belong to each object (e.g., a nucleus, cell, or organism). This task is known as *segmentation*. In our example (Figure 1), we wish to segment individual nuclei and individual DNA-damage-induced foci (in both cases to count them and measure their intensities), and we also wish to segment the cells to identify their borders and thus measure each cell's morphology. The first step toward segmentation is to distinguish foreground (objects of interest) from background. *Thresholding methods* [6] classify a pixel as foreground if it is brighter than a certain “threshold” intensity value. (Cells appear as bright objects on a dark background in fluorescent microscopy images. Other image types can use the same techniques by first *inverting* the image, turning dark regions into bright regions and vice versa.) Because of variations in staining and illumination, choosing a single threshold for all locations in all images is not always effective. Thus, the challenge is to determine appropriate threshold(s) automatically for each channel in each image. There are two main approaches to doing so:

*Global* thresholding algorithms compute a single threshold for each image. One method for global thresholding is by mixture models, which fit a mixture of two probability density functions (one for the foreground, one for the background) to the intensity histogram of the image, as illustrated in Figure 2. Mixture models work well when the histogram is clearly bimodal or when the mixture probability (the percentage of pixels that belong to the foreground) is known. Working with the logarithm of the intensities is often helpful because it can reduce the skewness of the intensity data. Another method, proposed by Otsu [7], chooses the threshold that minimizes the weighted sum of the intensity variance within each of the pixel classes (foreground and background). Otsu's method is often superior when the percentage of pixels belonging to the foreground varies substantially from image to image.

**Figure 3:**
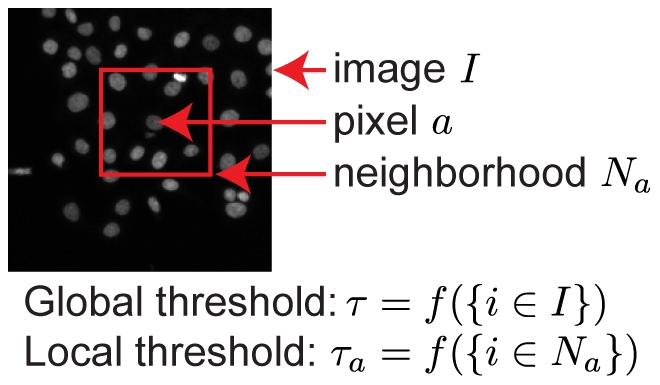
Local thresholding. Local thresholding methods compute the threshold *τ_a_* for a pixel *a* from statistics of intensities of pixels {i} in a neighborhood *N_a_* of *a* rather than from the entire image *I*. Original image from project described in Moffat et al. [80].

*Local* (a.k.a. *adaptive*) thresholding methods use different thresholds in different parts of each image, as illustrated in Figure 3. The threshold for a pixel is based on the intensity statistics of a local neighborhood rather than the entire image. Such methods are useful when the intensity of the background varies across the image due to uneven illumination or sample preparation. A danger with this approach is that if a part of the image contains tightly clustered objects (all foreground) or no objects (all background), the intensity statistics of the local area reflect only one class. One can detect that this condition occurs for a local neighborhood and instead interpolate from the thresholds of nearby pixels [8].

An alternative to local thresholding is to use global thresholding on images that have first been corrected for intensity variations in a separate preprocessing step known as *illumination correction* or *bias correction*. A smooth illumination function is fitted to the image, as described later. The intensity of each pixel is then adjusted by dividing by the value of the illumination function at that position. This adjustment improves segmentation; in our example assay, the slight decrease in lamp intensity at the edges of the images is barely noticeable by eye but causes dim foci there to be overlooked by automated algorithms.

**Figure 4:**
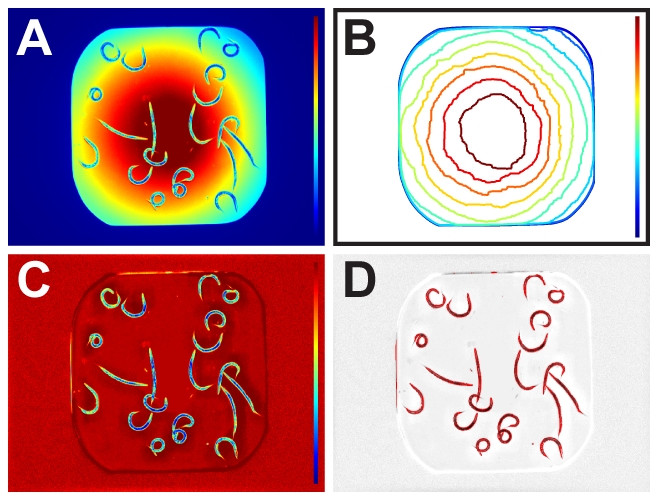
Illumination correction. (A) Brightfield image of *C. elegans* worms not amenable to thresholding because of intensity variations. The color bar on the right of the image shows that brighter pixels are displayed as red and dimmer pixels as blue. Original image from the project described by Moy et al. [32]. (B) Contour plot of smooth illumination function fitted to one or a set of images such as (A). (C) Corrected image obtained by pixel-wise division of (A) by (B). (D) Worms in the corrected image are consistently darker than the background and can therefore be identified by thresholding.

Figure 4 shows an example of illumination correction, using brightfield images of *C. elegans*, where the illumination patterns are more visible as compared to typical fluorescence images. This correction, followed by global thresholding, yields a result similar to that of local thresholding, but at a lower computational cost. The illumination function can be calculated by median filtering or by fitting a polynomial or spline surface, with the latter two being more resilient to overfitting and therefore more robust [9]. The function can be adjusted by placing more weight on pixels that are likely to be background. If the illumination variations are consistent between images in the set, fitting the illumination function to an average of several images, perhaps even the entire set, increases robustness.

Alternatives to thresholding are needed when the intensity inside the objects of interest is not markedly different from that of the background, as in many brightfield images. In these cases it is sometimes possible to classify pixels as foreground and background based on other features, such as local intensity variation or texture. It can be extremely difficult to choose *a priori* features that can identify the foreground; a more fruitful strategy has been to extract a large number of image features, hand-select some areas inside and outside the objects of interest, and use machine learning to find combinations of features that distinguish foreground from background [10],[11].

Noisy or low-contrast images can sometimes be handled more easily if assumptions can be made about the objects' shapes. For instance, the circular Hough and Radon transforms [12] can identify circular objects such as nuclei [13] and red blood cells [14], and spatial filters fit to a set of example objects can help identify similarly shaped objects by improving contrast [15]. Level-set methods, which constrain the objects' shape (among other properties) implicitly in the form of an energy functional, have proven effective for nuclei [16]. Identifying nuclei in tissue is much more difficult; some authors have reported success with template matching [17] or region-growing methods [18], while others have had to use manual seeding, where the researcher clicks once on each nucleus in the image [19].

## Splitting Clusters of Objects

**Figure 5:**
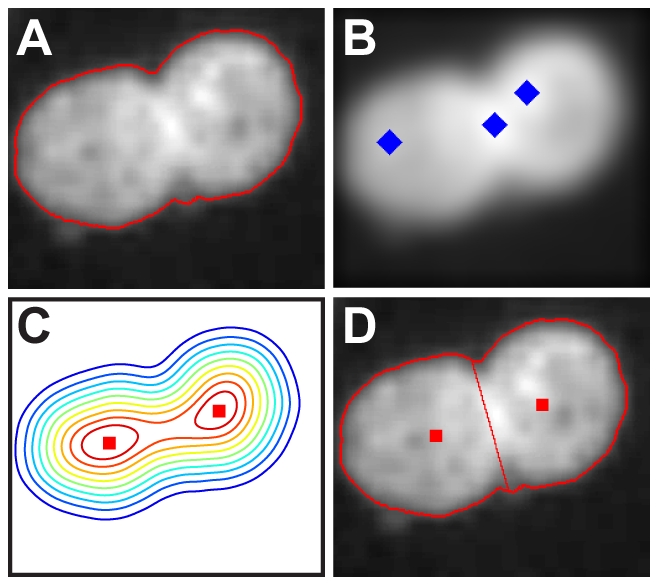
Splitting clusters of objects. (A) Thresholding this image of two nuclei results in one continuous outline rather than two objects. Original image from project described in Moffat et al. [80]. (B) The local maxima in the smoothed image correspond poorly with the centers of the nuclei. (C) The local maxima (red squares) in the distance transform of the image (shown as contours) correspond well with the centers of the nuclei. (D) Seeded watershed from the local maxima in (C) divides the cluster correctly.

Once foreground has been distinguished from background, additional processing is necessary to separate touching objects, as in Figure 5A. There are many algorithmic approaches to this problem; we describe here, as an example, a three-step process that is quite successful for many kinds of objects, including nuclei and DNA-damage-induced foci. The first step determines approximate centers of each object in the cluster. This can be accomplished in two ways, depending on the type of object: when objects are bright in the middle and dimmer towards the edges (the most common case for nuclei and DNA-damage-induced foci, both in fluorescent and brightfield images), identifying local intensity maxima in the smoothed image (Figure 5B) works well. When objects are not clearly brighter in the middle but quite round (commonly seen in brightfield images of yeast colonies), it is better to identify local maxima in the distance transform of the thresholded image (Figure 5C). The distance transform computes each pixel's value as the distance to the nearest background pixel, thus emphasizing indentations.

The second step identifies the dividing lines between touching objects using the “seeded watershed” algorithm [20]. The name of this algorithm alludes to the analogy of visualizing the image as a landscape. Each pixel's inverted intensity becomes its altitude in the landscape, so dark and bright regions become hills and valleys, respectively. Holes are drilled at each local minimum in the landscape (the “seeds”) and water pumped in at constant vertical speed. The points where the bodies of water meet are adopted as dividing lines between objects. This algorithm may be applied directly to images where the dividing lines between objects are dimmer than the objects, as is typically the case for nuclei and DNA-damage-induced foci. When no such dim dividing lines exist, as is typically the case for brightfield images of yeast colonies, the algorithm can instead be applied to the distance-transformed image, as shown in Figure 5D; then, the dividing line between the two objects is determined by their shape rather than by intensity changes between objects, and will usually be placed where indentations occur along the edge of the clumped objects [8].

The third and final step discards or merges objects based on models of what the objects should look like. For example, objects below a certain size can be discarded as debris, neighboring objects with similar intensities (or other relevant features) can be merged, or the watershed algorithm can be applied again to the distance-transformed image in order to break up remaining clusters [18].

## Identifying Subcellular Compartments

Cultured cells in high-throughput experiments are usually stained with multiple fluorescent markers, each of which labels a particular component or subcellular compartment of interest. Identifying the various subcellular compartments based on these stains is often required to obtain measurements that pertain to the biological process being studied. In our example, a DNA stain labels the nuclei; cytoskeletal markers, such as for actin or tubulin, label the cell overall; and the third channel is used for an antibody that stains DNA-damage-induced foci.

The nuclei of cultured cells can usually be identified with the illumination correction, thresholding, and declustering methods described in the previous sections because fluorescent DNA markers are specific and yield a good contrast between foreground and background. DNA-damage-induced foci are similarly analyzed, and given an appropriate marker, mitochondria, lysosomes, and other subcellular compartments can usually be identified by similar methods. Identifying the cytoplasm in fluorescent images poses a larger problem because the available markers (tubulin in our example) often yield low contrast and unclear boundaries between cells, depending on the cell type and culture conditions. An effective strategy has been to use region-growing methods (such as seeded watershed) to expand the cells around the previously identified nuclei [18]. A recent improvement uses not only the intensity gradient but also the distance to the nucleus to decide where to divide clustered cells [21]. It is sometimes unnecessary to precisely identify the cell boundaries: for instance, to determine whether a protein is predominantly in the nucleus or the cytoplasm, it can be sufficient to measure the average intensity of a protein in the nucleus and in a ring-shaped region around the nucleus, as a proxy for the cytoplasm.

## Measurements

Once cells and subcellular compartments are identified, they can easily be measured. While the following categories of general measurements are sufficient for most assays [22], measurements can also be designed for specific assays and applications [23]. Measuring a variety of cellular features beyond the primary readout/phenotype of interest is often useful for downstream categorization of samples, as we will see later.

### Counts

In our example assay, the primary readout of interest is the number of DNA-damage-induced foci per cell. The number of objects per image is often a useful readout in screening, even if only as a quality-control metric to ensure that cells have not been killed by the treatment.

### Size

The area of the image that is occupied by a cell, nucleus, DNA-damage-induced focus, or any other labeled cellular compartment can be measured. Keep in mind that the apparent two-dimensional *area* of an object may or may not be a good proxy for the *volume* of the object, depending on the cell type's growth characteristics (e.g., flat or spherical). In some cases, area may be a measure of cell attachment rather than cell size.

### Intensity

The intensity of a pixel is related to the amount of marker at that location. Thus, to a first approximation and assuming the depth of field is sufficient, the total intensity is proportional to the amount of substance labeled. For example, the total intensity of a DNA label in the nucleus can be used to identify cell-cycle phases based on DNA content—a relevant secondary readout for our DNA-damage screen. As another example, the intensity of a GFP reporter reflects the expression level of the fused gene. The maximal, minimal, mean, and integrated (total) intensity of each marker within each subcellular compartment can be measured, as well as correlation coefficients between channels, which are useful for capturing coexpression patterns (i.e., colocalization).

### Shape

There are a number of shape descriptors, each of which attempts to reduce some aspect of an object's shape to one or a few numbers. For instance, the ratio of the height and width of the smallest rectangle containing an object can serve as a measure of elongation, which may be of biological interest. As a measure of the object's compactness, one can use the squared perimeter divided by the area. Zernike shape features are also commonly used; they describe an object's shape in terms of the coefficients of a Zernike polynomial [24]. Shape measures such as these can be useful, for example, to distinguish apoptotic nuclei from normal, another relevant secondary phenotype for our DNA-damage screen.

### Texture

Texture descriptors characterize spatial smoothness and regularity for each marker, and are often useful for characterizing the fine patterns of localization of a protein. Texture measures fall into three general categories. Statistical texture descriptors, such as the moments of the intensity histogram (mean, variance, and so on), characterize textures as smooth, coarse, grainy, and so on. Structural texture measures describe arrangements of patterns such as stripes. Finally, spectral texture measures capture periodicity based on properties of the Fourier spectrum.

### Location

While the absolute location of a cultured cell within an image is usually meaningless, the distance from an organelle to the nucleus or to the cell membrane can be important. In time-lapse imaging, change in location over time is of interest [25].

### Clustering

The number of neighboring objects, the percent of the perimeter touching neighbor objects, and the distance to the nearest neighbor are measurements that characterize relationships between objects.

### Machine-Learning-Derived Measurements

Machine-learning algorithms have shown great efficacy in scoring samples based on sets of positive and negative controls [26]–[29]. This is because it is sometimes necessary to combine several measurements (among the features outlined above, for example) in order to classify a phenotype of interest versus controls. Hand-selecting such a combination of features can be difficult, especially when linear combinations are insufficient. Texture and shape features are particularly good examples of features that are difficult to use as direct readouts, but effective “raw material” for machine learning. End-user software tools (e.g., Definiens Cellenger [11] and our own open-source CellProfiler Analyst [30]) readily enable the application of machine learning algorithms for biological image analysis.

## Alternative Approaches

Despite the successes in this field, researchers often have images that are not readily tackled by applying algorithms in existing software. The typical workflow as described so far is effective for many, but certainly not all, two-dimensional images of cultured cells. Selecting algorithms and adjusting their parameters for a particular experiment can be daunting and time-consuming; the expertise of an experienced image analyst is often essential. Even with this assistance, many images remain intractable with ready-to-use software. Projects involving time-lapse or three-dimensional image sets, whole organisms, neuronal cell types, or brightfield images can be particularly difficult. Still, researchers have several options if existing software struggles to accurately identify and measure the objects of interest.

The first strategy is to adjust sample-preparation or image-acquisition techniques to make the images more tractable with existing software. Aside from the obvious good practice of consistent sample preparation and imaging (using automation where possible), a fix might involve changing staining concentrations, wash steps, or exposure times to improve the signal-to-noise ratio in the images. Using different staining or imaging techniques may also ease image analysis; for example, identifying nuclei from an unstained brightfield image is extraordinarily difficult, whereas adding a fluorescent DNA stain usually makes the identification of nuclei trivial.

Close collaboration is needed between the biologist who understands the goals and limitations of the experimental system and an image analysis expert who understands how algorithms will be affected by changes in the imaging protocol. For example, a computer scientist might suggest increasing exposure times without understanding the impact on the health of live cells in the experiment. Or a biologist might adjust staining concentrations in a way that makes structures more visible by eye but less tractable with a particular algorithm. Working together to optimize protocols for an experiment can yield vast improvements in the data, even in the absence of complex or customized algorithms. Some general principles for optimizing imaging experiments are discussed in Box 2.

**Box 2. Practicalities**

Novices are often surprised to find that they cannot rely on their eyes to choose sample preparation, image acquisition, and image storage techniques that are suitable for quantitative image analysis. The choices made can dramatically impact data quality, even though the effects of changing these protocols may not be noticeable by eye [74]. In this box we cover some of the basics; several tutorials are available in Box 1.

**Sample preparation:** Fundamentally, projects for quantitative image analysis should follow standard principles of good experimental design: positive and negative control samples should be included and all samples should be prepared and imaged in parallel under identical conditions. While this seems obvious, it is not unusual for researchers to mistakenly think it is appropriate to gather images from different experimental batches over the course of months, hoping to obtain quantitative relative measurements between them. Using lint-free materials and laminar flow hoods will avoid debris in samples, which can confuse image analysis algorithms. Parameters that should be tested for their effects on subsequent quantitative analysis include the selection of the labeling techniques (e.g., GFP-labeled proteins, fluorescent dyes, antibody staining), cell density, the concentration and timing of stains and fixatives, and time points. Multi-well plates are subject to “edge effects” where cells grow differently as a result of their well's spatial location on the plate. These problems can be avoided by not using the external rows and columns of the plate, or by incubating plates at room temperature after seeding [75].

**Sample formats:** Samples for imaging can be grown on coverslips, in live-cell chambers, on cell or tissue microarrays (usually with many thousands of samples per microscope slide [59],[76]), or in glass-bottom or optically clear plastic-bottom microplates (96, 384, or 1,536 wells per plate). For microscopes with laser-based autofocusing, black sidewalls work better than clear.

**Image acquisition consistency:** As is the case with sample preparation, the image acquisition parameters should also be kept as constant as possible. Lens magnification, auto-focus parameters, filter sets, and exposure times should be tested for their effects on subsequent image analysis. One common pitfall is having the microscope use an “auto-exposure” setting, thereby changing the exposure time from one image to another. This precludes quantitative comparison of the intensity of stains between images. Similarly, changes in lamp or filter settings between samples causes problems; we have noticed poor measurements due to fluctuations in power supplies that cause lamps to change their intensity transiently, failure to wait for a lamp to warm up (and thus stabilize its intensity) before collecting images, gradual loss of lamp brightness over its lifetime, and even unusually bright images due to the microscope room's door being opened partway through an experiment.

**Dynamic range:** The exposure time should be set such that the resulting images use a good proportion of the full dynamic range of the camera without saturating (overexposing) any images [77]. Most microscope-control software allows viewing a histogram of pixel intensities; the histogram should fill most of the available pixel intensities, but without the spike at the highest intensity that indicates saturation. Rescaling options that “stretch” each image's histogram should not be used, even though doing so inherently utilizes the full dynamic range, because this will preclude comparing intensities between images.

**Magnification, resolution, and binning:** In general, choosing a higher magnification lens produces higher resolution images (more pixels per µm^2^) that yield better quality image analysis. However, this comes at the cost of imaging fewer cells per field of view, which affects the statistical robustness of an assay. The optimal magnification is thus an empirical question. Binning combines light received by several adjacent pixels on the camera into a single pixel. This reduces the resolution of the images but increases the signal-to-noise ratio and the speed of image acquisition. The optimal choice for binning is also empirical.

**Illumination, bias, and background correction:** Many microscopes have an option to correct for uneven illumination in the field of view by a method called white-referencing or white-shading. In brief, an image of a “blank” field of view, called a white-reference image, is collected at the time of the experiment, and the pattern seen in that image is subtracted from each image that is collected. Assuming that this *a priori* correction is done correctly, it generally improves data quality. Some image-processing software has options for retrospective illumination correction when a white-reference image is not available.

**Sampling multiple fields of view:** The experimentalist should choose the number of images to acquire for each sample based on the statistics of the phenotype of interest. Dramatic effects can be readily detectable in a single image of each sample, whereas subtle or rare phenotypes might require dozens of images per sample to obtain statistically sound results. It is preferable to avoid capturing the edges of a well or coverslip within the field of view because these regions can confuse automated algorithms. Also keep in mind that cells can grow abnormally or clump in different locations within a well, so the choice between a central or peripheral location within the well depends on the characteristics of the cell type and the phenotype of interest.

**Image file format compatibility:** Many commercial manufacturers store images in their own proprietary file formats. When purchasing a microscope, preference should be given to those that enable saving images in a standard format that is easily readable by standard image analysis software. Efforts have been underway to standardize microscopy image file formats and metadata such as microscope settings. The most notable success in reading and converting microscopy images has been the open-source software BioFormats (http://www.loci.wisc.edu/ome/formats.html).

**Image file compression:** Some file formats (e.g., JPEG) compress the images in a “lossy” manner, meaning that image quality is sacrificed to reduce file size. These should be avoided for automated image analysis if at all possible. Not all compression is detrimental, however: some image-compression methods retain the original image information exactly, while reducing the size of the file. Such “lossless” formats (e.g., PNG and most TIFF formats) are perfectly acceptable for image analysis. Uncompressed file formats, such as BMP, are also a good choice for image analysis although the file size can be larger. A guide to file formats can be found on Wikipedia (http://en.wikipedia.org/wiki/Image_file_formats).

**Image file bit depth:** Bit depth describes the number of data bits used to represent the intensity value of a single pixel and is also known as *bits per pixel*. In other words, a file's bit depth indicates the number of separate grayscale intensity values (graylevels) that are allowable by the file format:

- 8-bit images have 2^8^ available pixel intensities, with a range of 0–255
- 12-bit images have 2^12^ available pixel intensities, with a range of 0–4,095
- 16-bit images have 2^16^ available pixel intensities, with a range of 0–65,535

Some microscope cameras capture 8-bit images and store them in 8-bit files, which all image-viewing software can display. Many other microscope cameras capture 12-bit images, which contain finer detail (in terms of graylevels) than 8-bit images. However, 12-bit file formats are incompatible with most software, so the alternatives are to save the image in an 8-bit format or a 16-bit format. Saving 12-bit image data in an 8-bit format is not ideal because the conversion will conflate intensity levels and thereby lose detail. A 16-bit format is thus preferable. Note that some image viewers can only handle 8-bit images, and some will display 16-bit images as very dark when they contain only 12-bit image data.

**Image file storage and retrieval:** Images are fairly large (compared to typical text or numerical data) and can be acquired rapidly; therefore data storage presents some issues to overcome, including procuring sufficient raw storage space and organizing the images with enough annotation to allow them to be readily retrieved later. This latter issue has been the focus of several groups [78],[79] (http://dough.ece.ucsb.edu/bisquik) as well as commercial electronic lab notebook offerings.

If, despite these efforts, images are still intractable to automated analysis but objects are readily visible by eye, it may be worth the investment to team up with computer scientists to develop a new algorithm (or identify one existing in the literature). Once validated, the algorithms can be added to existing open-source projects to give them a friendly user interface.

For many images, accurate identification and measurement of individual objects is impossible even by eye; for example, objects sometimes overlap or the borders between them are not visible. This is often the case in images of neuronal cells that intertwine amongst each other and images of tissues where cell boundaries are not distinctly visible. In some cases, measuring properties of the image as a whole can quantify the biological readout of interest. For example, segmenting individual *C. elegans* worms is often difficult because of clustering and severe illumination variation. Still, images with significant mortality can be detected based on the ratio of dead worm area to total worm area in the image. These values can be obtained by thresholding the inviability-stained images and the corresponding brightfield images, respectively [31],[32].

Another approach to images where accurate object identification cannot be achieved is to use machine learning, which can operate on measurements acquired from images without first identifying objects. For example, the WND-CHARM [27] algorithm constructs a classifier that can distinguish positive and negative control images based on arbitrary tiles of the images rather than identified objects. As in the machine-learning methods described above for foreground-background determination and object segmentation, the measurements used do not have to be specifically designed to target a particular biological phenotype of interest, but rather can be a general set of image measurements. Of course, results can sometimes be improved by adding measurements that are customized to the phenotype.

## Statistical Analysis

Statistical analysis is necessary in order to draw conclusions from the deluge of measurements in high-throughput imaging experiments. The end goal in our example screen is to rank-order samples by the number of DNA-damage-induced foci per cell and to identify which samples from the top and bottom of this list are statistically significantly different from controls. This step should include identification and elimination of systematic spatial artifacts. Spreadsheets, such as Microsoft Excel, are widely used because of their familiarity, although they are unable to handle large screening datasets and lack sophisticated analysis methods. High-throughput microscope vendors often bundle some data-analysis functionality with their instruments and image-analysis software. Investigators with knowledge of computer programming often write custom scripts, e.g., in Python, Matlab, or R. High-throughput screening software and general tools for multivariate analysis and visualization (e.g., GeneData Screener, SciTegic Pipeline Pilot, and SpotFire) have proven useful for image-based measurements, as have tools designed for flow cytometry or microarray informatics. However, such tools are often unable to display images linked to data, handle the huge datasets generated from images, or effectively handle the hierarchical structure of image-based measurements (since each image contains many objects). These features are gradually being added to commercial and open-source software, and tools specific to high-throughput image analysis data have also started to emerge. Two open-source examples are KNIME (http://www.knime.org) and our own CellProfiler Analyst [33].

## Conclusion

The field of biological image analysis continues to advance steadily, as computer scientists attempt to quantify ever more complex phenotypes in ever more challenging image types. In addition to fluorescence-microscopy images of cultured cells from screens, our example in this tutorial, researchers have been working towards accurate quantification of phenotypes in *C. elegans* and zebrafish. Other researchers are focused on algorithms for images derived from electron microscopy as well as from multi-spectral and multi-dimensional imaging. In parallel, the related field of biomedical image analysis continues to refine techniques for whole-animal and human organ and tissue imaging, including MRI and PET scans.

Historically, many of the techniques that are useful for biological images were first developed for other purposes such as face recognition, satellite surveillance, and manufacturing quality control. This trend will likely continue, as the field transitions to rely more heavily on machine-learning techniques, for example. A more practical but very welcome development in the field has been the increasing compatibility among various image-acquisition and image-processing software packages. As this software becomes more modular and interoperability between systems improves, bench biologists benefit by spending less time shepherding data from one package to another and more time designing and interpreting their experiments.

We have only seen the tip of the iceberg in terms of extracting maximal knowledge from biological images. Currently, the goal is typically limited to measuring a precise biological phenotype at hand to address a well-constrained biological question. Yet the richness of information present in images lends itself to less-biased approaches. This is exemplified by efforts to catalog protein-location information for large numbers of human genes [34]. That particular field has progressed from a biologist using words to describe the appearance of a protein's staining pattern (at best using a controlled vocabulary) and subjective selection of a typical example cell to using quantitative measurements of many cells to objectively select the most typical example cells for publication or display. Even further, quantitative data are now being used to produce generative models of each protein's location. Reducing a visual pattern to a generative model enables patterns to be quantitatively compared to each other. This enables identifying samples that yield similar patterns as well as novel patterns [26], [35]–[39]. Excellent opportunities exist for using quantitative image-derived data in systems-biology research to gain a global view of the relationships between genes. This data source is so far largely untapped.

The demand for accurate image analysis in biology continues to grow. Given the prevalence of automated microscopes, large-scale experiments are becoming more routine, and even small-scale experiments are producing more data than before: time-lapse images, for example, can be readily captured and lend a rich source of dynamic information about biological systems. Automatic image analysis benefits biology by enabling quantitative readouts for microscopy, especially for high-throughput experiments. Although image analysis is a large field of study, and myriad methods have been developed for particular purposes, an understanding of the basic concepts and techniques will enable modern biology researchers to better design and carry out quantitative image analysis, skills that are likely to be increasingly necessary as microscopy automation becomes widespread in biological laboratories.
